# Food safety and sanitation challenges of public university students in a developing country

**DOI:** 10.1002/fsn3.2399

**Published:** 2021-06-14

**Authors:** Kevin Serrem, Csaba Bálint Illés, Charlotte Serrem, Bridget Atubukha, Anna Dunay

**Affiliations:** ^1^ Szent Istvan Campus Hungarian University of Agriculture and Life Sciences Gödöllő Hungary; ^2^ Department of Consumer Sciences School of Agriculture and Biotechnology University of Eldoret Eldoret Kenya; ^3^ Faculty of Bioscience Engineering Katholieke Universitiet Leuven Leuven Belgium

**Keywords:** attitude, food borne illnesses, food safety and sanitation, knowledge, practice, university students

## Abstract

Inadequate catering facilities in Kenyan public universities compel students to handle and prepare their own food, in environments not designated for food preparation such as rooms in hostels. This study investigated the level of food safety and sanitation knowledge, attitude, and practice, among students in an effort to prevent food‐borne diseases. A cross‐sectional study was conducted among 535 students from two public universities in Kenya. Data were obtained through a piloted, structured questionnaire in line with Food and Agriculture Organization (FAO) guidelines, administered to students from seven different departments. Eighty percent of the students had adequate levels of food safety and hygiene knowledge, while 70% had a positive attitude toward food safety and sanitation. An average of 74% engaged in inadequate food safety and hygiene practices, with majority citing lack of equipment as a major contributor. ANOVA results revealed significant correlation between the gender and knowledge and practice of food safety and sanitation (*F* = 30.328, *ρ* = 0.000) and (*F* = 18.177,*ρ* = 0.000), respectively. Binary logistic regression showed that knowledge (β = 3.677, *p* < .000) fostered the practice of food safety and sanitation more in comparison with attitude (β = 2.244, *p* < .000). Kenyan universities should consider introducing food safety courses that emphasize Food Safety Management System (FSMS) and Hazard Analysis Critical Control Point (HACCP) practices and procedures especially to non‐science‐based courses, in addition to providing students with proper cooking and food handling facilities.

## Graphical abstract

The study that was conducted among university students in Kenya revealed that the university students have adequate knowledge levels, toward food safety and sanitation, but they have a less satisfactory attitude level.Despite knowledge being the biggest contributor to the practice of food safety and sanitation, many students fail to practice proper food safety and sanitation, which may lead to food safety and food quality problems.

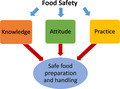

## 1 INTRODUCTION

Food‐borne diseases are an increasing public health problem responsible for considerable morbidity and mortality (Linscott, 2011). An estimated 600 million people globally fall ill after consuming contaminated food and 420,000 lose their lives daily, resulting in at least 33 million deaths of healthy people every year (King et al., 2017; Szakály et al., 2020). In developing countries, the occurrence of food‐borne diseases is aggravated by poor levels of hygiene, lack of adequate clean drinking water, contaminated and inappropriate food storage facilities, lack of food safety education, contaminated equipment, food from unsafe sources, and inadequate cooking FAO et al.(2018); (Lynch et al., 2006). Additionally, with the current ongoing Corona virus (COVID‐19) pandemic, food handlers and consumers are at higher risk than ever before from infection through contact with kitchen surfaces and foods, handled by infected persons (Liu et al., 2020; World Health Organization, 2020). Consequently, governments in developing countries and policy makers require global efforts in collaborations, funding, awareness, and commitment to managing this problem.

Among the causes of food borne illness, is the consumer's inclination toward a food and eating place exposing them to innumerable risk (Adam et al., 2014; Medus et al., 2010). The risks include material products consumed, behavior and attitude of the food service establishment employees, and the environment (tangible feature at the time of purchase) in which the food is prepared and served (Goh et al., 2013). Therefore, the eating environment is a benchmark for establishing whether a food service outlet provides safe foods or not (Worsfold & Worsfold, 2008; Tóth & Bittsánszly, 2014; Szakály et al., 2020).

As numerous studies have indicated, their knowledge and practical skills in food safety topics are limited, hence they jeopardize their health by putting themselves at risk of contracting food borne illnesses (Byrd‐Bredbenner et al., 2007; Ferk et al., 2016; Green & Knechtges, 2015; Stratev et al., 2017). Of particular concern in university settings is that a majority of college students prepare their own meals, some for the first time in life and many engage in behavior that places them at risk due to poor food handling and holding temperature practices (Al‐Shabib et al., 2016). According to Byrd‐Bredbenner et al. (2008), students frequently eat food considered, either raw or undercooked, of animal origin or whatever is convenient. Male students violated food safety and sanitation rules and regulations most, rendering them eligible for food safety training to increase knowledge (Byrd‐Bredbenner et al., 2007). Hence, it is crucial to determine the food safety knowledge, attitude, and practice of college students to have a basis for reducing their risk of contracting food‐borne diseases.

Catering facilities in Kenyan public universities have been tasked with provision of subsidized, wholesome foods and beverages, affordable to the entire student fraternity (Were, 2017). However, a majority of such facilities are faced with massive challenges such as low financial output, escalated production costs, low levels of client satisfaction due to repetitive menus, untimely food shortages, and unreliability on food variety (Were, 2017). Consequently, students are compelled to facilitate their own food preparation and consumption. Hence, students are exposed to food borne disease when they prepare meals in places not designated for meal preparation, such as hostel rooms (Mbirithi, 2013). There is limited information on Kenyan university students' level of knowledge, attitude, and practice of food safety and sanitation. Additionally, how their knowledge and attitude of food safety and hygiene, influences practice. Therefore, with the use of piloted questionnaires, the study investigated the level of knowledge, attitude, and practice of food safety and sanitation among Kenyan university students and the influence their knowledge and attitude of food safety and sanitation had on practice.

## 2 MATERIALS AND METHODS

### 2.1 Research design

The study was conducted in two public universities in Western Kenya. A cross‐sectional study was used to assess the level of knowledge, attitude, and practice of food safety among university students. A total of 535 students from the two universities taking courses in the departments of Consumer Science, Business Management, Civil Engineering, Education technology, Environmental Studies, Human Resource Management, Natural Resources, and Anthropology were selected for the study. The target population was students who lived on campus in the university hostels. Purposive judgmental sampling was used to select the two public universities out of a total of 23 public universities found in Kenya. Since they were public institutions, the researcher had prior knowledge that the institutions had a number of similarities. First, students were subjected to and lived under similar conditions; second, the universities shared a variety of courses, hence purposive judgmental sampling was also used to select particular departments.

Multistage sampling was used to select respondents. The first stage involved stratification of respondents into the various departments; secondly, proportional allocation was conducted from various years available within departments, and lastly, systematic random sampling was used to select particular respondents. Questionnaires were administered to the respective classes through the help of chosen departments, which contacted particular class representatives, who were then issued with the questionnaires. The students filled questionnaires either before or after lectures, as they took approximately 10–15 min to answer. Filled questionnaires were immediately taken back to respective departments. Before data collection began, a pilot study was conducted on 100 students of a technical college. This greatly assisted in restructuring and clarifying questions in the questionnaire making them easy to understand. The questionnaire was again retested and reviewed by two experts. The data collection period lasted 2 weeks. Out of a total of 600 questionnaires distributed, only 535 were available and viable for analysis, hence, 89% participated in the study.

### 2.2 Questionnaire

**FIGURE 1:**
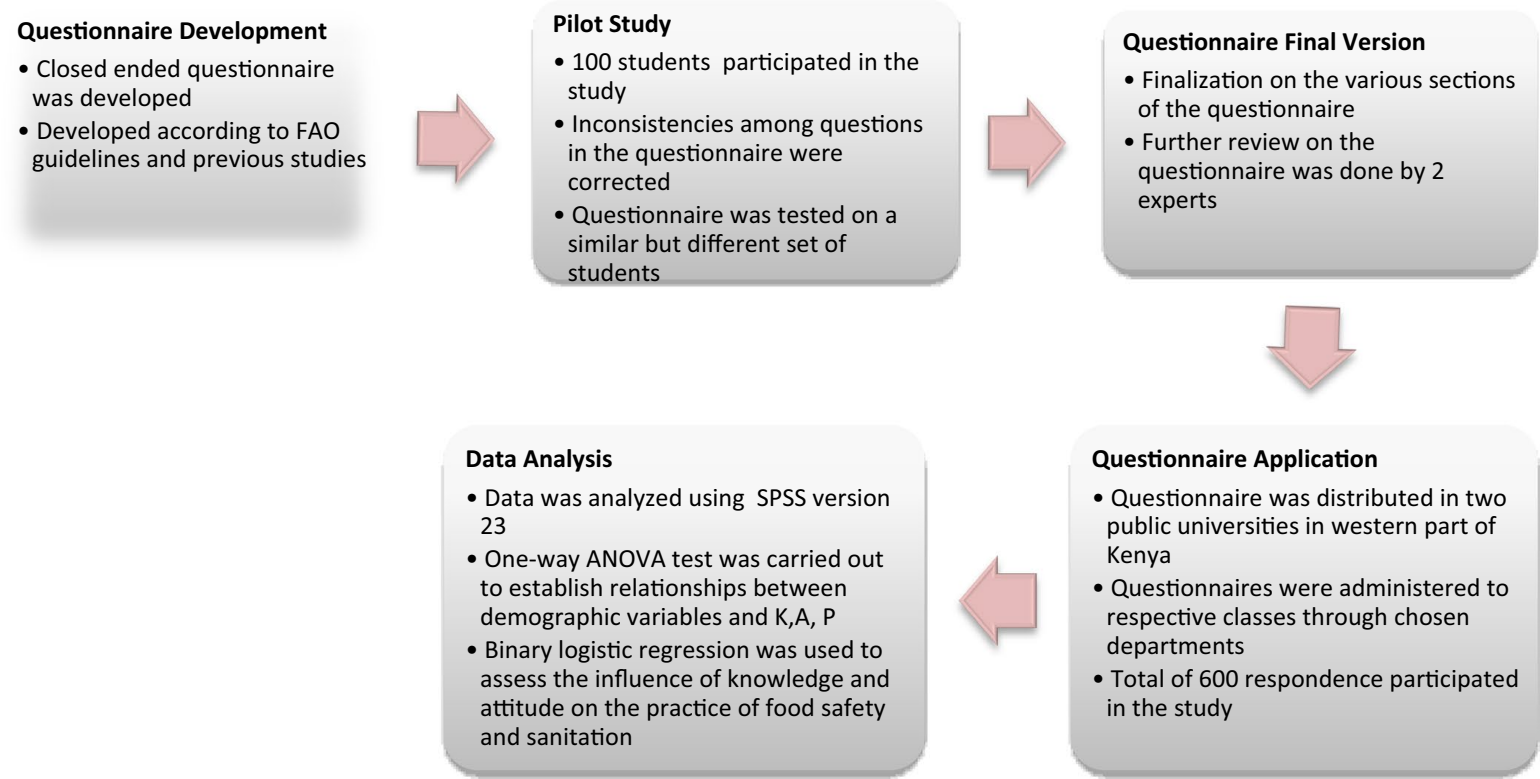
Questionnaire development and application process

The questionnaire which was developed, in line with the preventive measures laid down by FAO, IFAD, UNICEF, WFP, and WHO (2018), and previous studies (Al‐Shabib et al., 2016; Ferket al., 2016, and Adam et al., 2014) was divided into four sections. Section A included demographic data of respondents' age, gender, nationality, and college of study. Section B addressed food safety knowledge that was assessed using 13 items. Each scored 1, if the answer was right, and 0 for the wrong answer or I don't know. The total score ranged from 0 to 13 and a high score indicated a high level of knowledge on the topic. According to Al‐Shabib et al., 2016; Ferket al., 2016, and Adam et al., 2014, success was ranked at 100%–80% and above, while average was ranked 70% –40% and failure at 30%–0. Therefore, results were compared against a similar performance scale, which was indicated as 0–4 aslow/failure, 5–8 as average/pass, and 9–13as high/successful, Section C, is where food safety attitudes were assessed using twelve items. Each item consisted of five levels with a score ranging from 1 to 5, which implied “Strongly disagree” to “Strongly agree.” The total score for this section ranged from 12 to 60, and a high score indicated more concern about food safety. Results were equally comparable to a performance scale that indicated 12–20 as low/ failure, 21–40 as average, and 41–60 as high. Section D assessed the food safety practices using twelve items. Participants were asked to score according to the frequency of these practices: one = never; two = occasionally; three = sometimes; four = often; and five = always. The total score for these items ranged from 12 to 60 and a high score indicated good food safety practices. Likewise, results were compared with a performance scale, which was rated 12–20 as low, 21–40 average, and 41–60 high. Respondents participated in the study at their own free will, and were instructed not to write their names or identities on the questionnaire. This was to ensure anonymity and reduce respondent bias. In order to determine the construction and content validity, two food safety experts reviewed the questionnaire before administering it (Figure 1).

### 2.3 Data analysis

Data were analyzed using SPSS Software for Windows Version 23.0. Data were presented in means and frequencies (%). A one‐way ANOVA test was carried out to establish relationships between demographic variables and knowledge, attitude, and practice. *p*‐value of less than .005 was considered statistically significant. Binary logistic regression was used to assess the influence of knowledge and attitude on the practice of food safety and sanitation. During data analysis, data from both universities were analyzed together since both universities exposed their student to similar conditions.

### 2.4 Ethical approval

Ethical approval was granted by the National Commission for Science, Technology and Innovation, (NACOSTI) in Kenya permit number, NACOSTI/P/1981086/28440. Participation in the study was voluntary, and respondents were assured of anonymity and utmost confidentiality of the information provided.

## 3 RESULTS

### 3.1 Demographic profile of respondents

Table 1 shows the demographic characteristics of the respondents. Out of the 535 public university students who participated in the study the males (49.6%) were almost equal to the females (50.4%). A high percentage of respondents (64.1%) was aged between 20 and 24 years, with only 6.7% aged 25 and 29 years. Most of the students were single (52.7%), while only 13.5% were married.

**TABLE 1:** Profile of Kenyan public universities student respondents

Demographics characteristics	*N*	Percentage
Gender		
Men	265	49.6
Women	270	50.4
Age		
19	96	17.9
20–24	343	64.1
25–29	36	6.7
30–34	60	11.2
Marital status		
In a relationship	181	33.8
Married	72	13.5
Single	282	52.7

### 3.2 Knowledge of food safety

The mean partial knowledge scores for 13 knowledge items are shown in Table 2. Only, a few (62%) participants did not know that wearing jewelry such as watches, earrings, and rings might lead to the contamination of food. Equally, another dismal percentage (7%) of participants demonstrated inadequate knowledge on the suitable temperature for growth of microorganisms. Additionally, only 67% of respondents confirmed insufficient knowledge on the immediate refrigeration of purchased perishable food items (meat, vegetables) before use, while majority of the respondents 99%, 94%, and 91% had adequate knowledge that utensils should be clean before preparing food, storage of cooked and uncooked food in similar containers in a refrigerator, and the use of tasting as a way of checking sufficiency of cooking, respectively. An average of 82% of the students indicated scores that were classified as “high” for all knowledge items.

**TABLE 2:** Partial knowledge scores of university students about food safety and sanitation (*n* = 535)

Knowledge Items	Percentage %	Frequency (*N* = 535)
Consumers are responsible for food and safety after purchase of the food	82	438
Utensils should be clean before preparing food	99	529
Cooked and uncooked food should be kept in the same container of the refrigerator	94	503
After using a chopping board to cut meat, you need to wash it with warm water and soap before cutting vegetables	89	476
One cloth is enough in the kitchen, to wipe hands, dishes, and the work bench	85	455
Tasting is the best method to check whether the cooking was sufficient	91	487
Do you refrigerate purchased perishable food items (meat, vegetables) before use	67	358
Most suitable temperature for growth of microorganisms is between 30 and 40°C	65	348
Damage in food packing can cause food spoilage	84	449
Food with bad smell, appearance, or taste is unsuitable for consumption	90	481
Hand washing before handling food reduces the risk of contamination	89	476
Using watches, earrings, and rings allows food to be contaminated	62	332
It is alright to handle food despite an injury on your hands allows food to be contaminated	73	390

### 3.3 Attitude toward food safety

The findings on attitude regarding proper food safety and sanitation are shown in Table 3. Percentage scores for the 12 items scale developed to assess attitude related to food safety are represented in Table 3.A Cronbach alpha of 0.75 indicated that all items had a negligible impact; hence, all items were incorporated in to the scale. The mean scores for all items are high indicating that respondents either agreed or strongly agreed with each of the items. Six of the twelve items received ratings higher than 80%. These include concerns about food safety incidents in recent years in their county (89%), ensuring cleanliness of food purchased (88%), reheating leftover foods before consumption (85%), willingness to change inappropriate food safety practices (84%), and the current situation of food safety in the school canteen (83%). All these clearly indicate university students' concerns about food handling by various food outlets. This is also important now due to the corona virus pandemic. On the other hand, study participants scored poorly on items such as the importance of frequent hand washing to ensure safety in food consumed (54%) and smoking during food preparation (69%).

**TABLE 3:** Partial attitude scores of university students about food hygiene and safety (*n* = 535)

Attitude Items	Percentage%	Frequency (*N* = 535)
Leftover foods should be reheated before consumption	85	454
I shouldn't always consider freshness of food during purchase	36	192
I should ensure cleanliness of food purchased	88	470
It is okay to smoke during food preparation	69	369
Food contact surfaces should be cleaned using sanitizing agents	76	406
It is not a must to wash hands frequently to ensure safety of food consumed	54	289
Are you concerned about the food safety incidents experienced in your country in the near past	89	476
You are willing to change your inappropriate food safety practices	84	449
Are you concerned about the food safety situation currently being experienced in your country	74	395
You are concerned about the safety of food in restaurants around the school	84	449
You are concerned about the current situation of food safety in the school canteen	83	444
You are willing to improve your knowledge of food safety	76	406

### 3.4 Practices toward food safety

The results in Table 4 indicate that the majority of respondents (89% and 85%) ensured that they always use washed chopping boards and knives and also ensured to check the expiry date of food items before use. Additionally, only 76% of the students managed to store raw foods away from cooked foods and avoided the use of expired foods, while a further 74% of the participants remembered to wash their hands before touching food. On the other hand, 69% of the respondents rarely checked the cleanliness of utensils before cooking or washed utensils with warm water. Additionally, 56% of the participants do not store their leftovers in a refrigerator, while 64% of the students rarely remove watches, rings, and jewelry before cooking.

**TABLE 4:** Partial practice scores of university students about food hygiene and safety (*n* = 535)

Practice Items	Percentage%	Frequency (*N* = 535)
I will ensure to use a washed chopping boards/ knifes	89	476
I will ensure to wash my hands before touching food	74	396
I will ensure not to use food from damaged packages	75	401
I will ensure food is well cooked	84	449
I will ensure to check the expiry date of food items before use	85	456
I will clean the kitchen counter and utensils after food preparation	72	385
I would prepare food while having wounds bruises or injuries	84	449
I will store raw food away from the cooked food	70	374
Hardly do I use expired food	76	406
Hardly do I store leftover foods in the refrigerator	56	300
Hardly do I check the cleanliness of utensils before cooking	69	369
Hardly do I wash dishes with warm water	69	369
Hardly do I remove watches, rings, and jewelry before cooking	64	342

### 3.5 Demographics in relation to knowledge, attitude, and practice

#### 3.5.1 Knowledge

The results in Table 5 indicated that female students had more knowledge on food safety and sanitation, which was slightly higher (85%) than that of their male counterparts(79.2%), revealing that there was a significant correlation between gender and knowledge in food sanitation (*F* = 30.328, *ρ* = 0.000). Furthermore, students aged 25–29 years were most knowledgeable (83.3%) on food safety and sanitation compared with all other age groups who showed that there was no significant correlation between age and knowledge in food sanitation (*F* = 1.225, *ρ* = 0.300).

**TABLE 5:** Demographics in relation to knowledge, attitude, and practice

	Knowledge				Attitude			Practice		
	Descriptive statistics		ANOVA		Descriptive statistics	ANOVA		Descriptive statistics	ANOVA	
	*N*	Mean (*S.D*)	*F*	Sig.	Mean (*S.D*)	*F*	Sig.	Mean (*S.D*)	*F*	Sig.
Gender										
Female	270	0.85 (0.10)	0.24	0.00	0.76 (0.23)	1.30	0.26	0.80 (0.13)	18.8	0.00
Male	265	0.79 (0.12)			0.74 (0.25)			0.73 (0.19)		
Total	535	0.82 (0.12)			0.75 (0.24)			0.77 (0.17)		
Age										
Under 19 years	96	0.80 (0.14)	1.23	0.30	0.76 (0.17)	7.47	0.00	0.73 (0.16)	1.77	0.15
20–24 years	343	0.82 (0.09)			0.77 (0.24)			0.77 (0.15)		
25–29years	36	0.83 (0.13)			0.73 (0.24)			0.78 (0.11)		
30–34 years	60	0.82 (0.17)			0.58 (0.28)			0.76 (0.28)		
Total	535	0.82 (0.12)			0.75 (0.24)			0.77 (0.17)		
Marital status										
In a relationship	181	0.81 (0.12)	2.86	0.06	0.74 (0.27)	0.30	0.74	0.73 (0.16)	9.13	0.00
Married	72	0.80 (0.15)			0.77 (0.27)			0.81 (0.23)		
Single	282	0.83 (0.10)			0.75 (0.21)			0.78 (0.15)		
Total	535	0.82 (0.12)			0.75 (0.24)			0.77 (0.17)		
Course										
Family and Consumer Science	90	1.00 (0.00)	4.17	0.00	1.00 (0.00)	4.17	0.00	0.79 (0.17)	4.64	0.00
Business Management	120	0.99 (0.09)			0.99 (0.09)			0.75 (0.21)		
Civil Engineering	30	1.00 (0.00)			1.00 (0.00)			0.79 (0.07)		
Education Technology	48	0.90 (0.31)			0.90 (0.31)			0.76 (0.10)		
Environmental studies	43	1.00 (0.00)			1.00 (0.00)			0.81 (0.11)		
Human Resource Development	48	1.00 (0.00)			1.00 (0.00)			0.69 (0.11)		
Natural Resources	30	1.00 (0.00)			1.00 (0.00)			0.86 (0.06)		
Anthropology	60	1.00 (0.00)			1.00 (0.00)			0.80 (0.17)		
Economics Studies	66	0.94 (0.24)			0.94 (0.24)			0.70 (0.20)		
Total	535	0.98 (0.14)			0.98 (0.14)			0.77 (0.17)		

Note

Data presented as mean and *S.D*; one‐way ANOVA used test the correlation between demographic variables against; knowledge, attitude, and practice.

With regard to relationship status, a majority (53%) of the participants were single, while a slightly lower (34%) percentage was married. The study further showed that single students were most knowledgeable (83%) in comparison with their colleagues who were either married (80%) or in a relationship (81%). On the other hand, ANOVA results yielded no statistically significant relationship between marital status and student knowledge in food sanitation (*F* = 2.862, *ρ* = 0.058). Results showed that students who took science courses demonstrated higher levels of knowledge (99%) in relation to food safety and sanitation. These included courses such as Consumer Science, Environmental Studies, Natural Resources, and Civil Engineering. In contrast, students in Art Courses exhibited slightly lower levels of knowledge compared with students in courses such as Education technology and Economics studies scoring 90%and 94%, respectively. Also, ANOVA results yielded a positive correlation between the science courses and knowledge in food sanitation (*F* = 4.17, *p* = .00).

#### 3.5.2 Attitude

As viewed in Table 5, the results affirmed that female students, who also doubled up as majority, had a more positive attitude (76%) toward food safety and sanitation than male students who recorded a slightly lower value of S 74%. However, there was no significant correlation between the gender and attitude in food safety and sanitation (*F* = 1.297, *ρ* = 0.255). Moreover, students in the ages of 20–24 years had a slightly more positive attitude (77%) toward food safety and sanitation as opposed to their counterparts who were under 19 years (76%), 30–34 years (73%), and 25–29 years (58%). These further implied that there was a significant correlation between the age and attitude toward food safety and sanitation (*F* = 7.466, *ρ* = 0.000).

Despite the married participants indicating the highest levels (76.5%) of attitude toward food safety and sanitation, ANOVA results yielded no significant relationship between marital status and student attitude in food safety and sanitation (*F* = 0.296, *ρ* = 0.744). Nonetheless, a majority of the students who studied science‐related courses demonstrated high (100%) levels of positive attitudes toward food sanitation. This included courses such as Consumer Science, Environmental Studies, Natural Resources, and Civil Engineering. In comparison, students in Art courses such as Education (90%), and Economic Studies (94%) showed slightly lower levels of attitudes. These illustrated a positive correlation between the scientific courses and food sanitation (*F* = 4.17, *p* = .00).

#### 3.5.3 Practice

Table 5 shows the demographic characteristics of respondents in relation to practice. There was a significant correlation between gender and practice of food safety and sanitation (*F* = 18.177, *ρ* = 0.000). Female students had more practice (80%) of food safety and sanitation than males (73.4%). Furthermore, students aged 25–29 years were more willing to practice (78.2%) food sanitation as opposed to those aged 20–24 years (77.3%), 30–34 years (76.2%), and under 19 years (73.1%) showing no significant correlation between age and practice of food sanitation (*F* = 1.773, *ρ* = 0.151). On the other hand, the study demonstrated that most married students practiced food safety (80%) in comparison with all other marital status. Similarly, to knowledge and attitude, majority of the students enrolled in science courses were more cautious about food safety and sanitation hence tended to practice more food safety and sanitation. The respondents' results at Natural Resources (86%), Environmental Studies (81%), Consumer Science (80%), Anthropology (79%), and Civil Engineering (79%) fields of study show this. Students doing art courses such as Business Management (75%), Economics Studies (70%), and Human Resource (69%) were less cautious.

### 3.6 Binary logistic regression on the influence of knowledge and attitude on practice

Binary logistic regression was used to determine the influence of knowledge and attitude on the practice of food safety and sanitation, of Kenyan university students. The characteristics of the output are shown in Table 6. They include the Exp (B), which represents the odds ratio (likelihood ratio), the Wald value, and the significance (*p*) which shows the degree of importance the individual predictor has on the entire model, B and the SE that represents the unstandardized beta and standard error, respectively. To be considered significant to the model, a predictor variable should have a combined odds ratio value of more than 1 and a significant (*p*) value of less than .05 (Kinnear & Gray, 1999; Pallant and Bailey, 2005). When the Exp (B) or odds ratio is less than 1, increasing values of the variable correspond to decreasing odds of the event's occurrence and vice versa.

**TABLE 6:** Binary logistic regression on influence of knowledge and attitude on the practice of food safety and hygiene/ choice of eating place

	B	S.E.	Wald	Df	Sig.	Exp (B)
Knowledge1	3.677	0.559	43.332	1	0.00	39.544
Attitude1	2.244	0.468	22.995	1	0.00	9.433
Constant	−2.439	0.495	24.241	1	0.00	0.087
Model Summary						
−2 Log likelihood	184.338a					
Cox & Snell R square	0.306					
Nagelkerke R square	0.602					
Hosmer and Lemeshow test						
Chi‐square	1.471					
Df	1					
Sig.	0.225					
Omnibus tests of model coefficients						
Chi‐square	195.336					
Df	2					
Sig.	0.000					

The model predicted 30.6%–60.2% of the variation in students' knowledge and attitude of food hygiene and sanitation and its influence on practice indicated by the Cox & Snell R Square 0.306 and Nagelkerke R Square 0.602. The model emerged as a good predictor of students' influence of knowledge and attitude on practice of food safety and sanitation. This was further confirmed by the Omnibus Tests of Model Coefficients (chi‐square, 195.336, *p* > .000). The findings indicated that knowledge (β = 3.677, *p* < .000) and attitude (β = 2.244, *p* < .000) significantly influenced the practice of food safety and sanitation among Kenyan university students. The greatest significant influence of the practice of food hygiene and sanitation was exerted by knowledge. Consequently, students with adequate knowledge of food hygiene and sanitation were significantly more (39.5 times) likely to practice it, compared with a likely hood of (9.4 times) exerted by attitude.

## 4 DISCUSSION

As alleged earlier, a majority of Kenyan universities lack the capacity to sustainably provide affordable meals to the growing number of students. Students without adequate training on food handling and preparation other than what is taught at home have to prepare meals for themselves in hostel rooms, which lack necessary facilities for food preparation. During the current COVID‐19 pandemic, this poses a higher risk to such students, as students lack proper food handling and preparation facilities to maintain basic HACCP procedures. The study revealed an adequately high (80%) level of knowledge, among Kenyan university students, concerning food safety and sanitation. This is comparable to the findings of Byrd‐Bredbenner et al., (2007) and Sharif and Al‐Malki (2010), where more than 60% of sophomore students demonstrated high levels of knowledge regarding practices related to cleanliness of kitchen utensils and kitchen surfaces, prevention of cross‐contamination, and hand hygiene. Training has been shown to improve knowledge and practice. For example, Ncube et al. (2020) demonstrated that food handlers in Zimbabwe had high (94%) food safety and sanitation knowledge after training. A similar study by Yusof fetal (2018) also reported high (86.7%) knowledge scores with regard to food poisoning among students of dietetics in a public university in Malaysia after training.

Despite having sufficient knowledge in food safety and sanitation, students were limited in knowledge in some critical sections of food safety, such as temperature control and wearing of jewelry during food preparation, where students scored 65% and 62%, respectively. These findings are in line with Tirado and Schmidt (2000) who found that crucial food handling knowledge is on the decline among college students, as 45.6% of food borne illnesses are due to temperature abuses during food processing, poor refrigeration, and inappropriate storage temperatures of leftover or recently cooked meals. Comparable results were reported by Yusofetal (2018) and Nderitu et al. (2020), as dietetics students in Malaysia only managed to only score (37.7%) in relation to the adequate temperature for reheating leftover food. Another study by Al‐Shabib et al. (2016) reported that only 50% of students in a Saudi Arabian university removed watches, rings, and jewelry before cooking. On the contrary, Ncube et al. (2020) noted that more than 89% of the food handlers in Zimbabwe were aware that the wearing of earing rings, watches, and necklaces significantly increased the risk of food contamination.

The study disclosed that female students were more knowledgeable compared with their male counterparts. These findings are similar to results by Yusof et al. (2018), where female students were significantly more knowledgeable. Nkhebenyane and Lues (2020) cited comparable results, after carrying out similar studies in Hospices in South Africa. On the contrary, Unklesbay et al. (1998) found no significant difference between gender scores as both were enrolled in the same food safety course. Student should be aware of the necessity of adequately washing and sanitizing hands before coming in to contact with food, proper washing and sanitization of utensils and food preparation surfaces, and proper washing of food items especially uncooked foods such as fruits. According to World Health Organization (2020), despite COVID‐19 being a respiratory diseases requiring human‐to‐human contact, respiratory droplets from infected persons will settle on various surfaces and be transmitted through the mouth, nose, and eyes, through touching the face or consumption of an uncooked food item containing the virus.

With regard to attitude, majority of the students (85%) were in accord, that it, was paramount to reheat leftover food before consumption. Similar results were also echoed by Ncube et al. (2020), where by more than 89% of food handlers were in agreement that leftovers should be adequately reheated so as to prevent food poisoning. Likewise, a study by Al‐Shabib et al. (2016) established that due to the poor knowledge and negligible attitude toward temperature control almost half (48.5%) of the students failed to regulate temperatures on their refrigerators, which led to the storage of food in unsafe temperatures and ultimately, failure in reheating leftovers to adequate temperatures for the right before consumption.

The studies revealed that a majority of the students did not mind smoking while preparing food which in retrospect leads to food contamination. This sharply contrasts with the study of Ncube et al. (2020), where food handlers were aware of the fact that eating, drinking, and smoking while preparing or serving food, increased food contamination risk. In line with the findings of various authors (Bolton et al., 2008, Martins et al., 2012 and Sani & Siow, 2014), it has been established that hand washing is pivotal in prevention against food contamination by removing harmful bacteria such as *E*. *coli*, *Salmonella*, and *Staphylococcus* *aureus* on the hands of food handlers. Additionally, according to the European Food Safety Authority (EFSA) (2020), the human corona virus 229E (HuCoV‐229E) survived on surfaces used during food preparation. Such surfaces include polyvinyl chloride, polyfluorotetraethylene, glass, ceramic tiles, and stainless steel for 5 days and on silicon rubber surfaces at 21°C with a relative humidity of 30%–40% for 3 days. Hence, it is important for food handlers to always wash hands and sanitize food surfaces.

In practice, a significant number of Kenyan university students both prepared and served foods with wounds, bruises, or injuries. This is in line with a similar study by Nkhebenyane and Lues (2020) on South African food handlers where an equally significant majority of food handlers concurred with the fact that wounds pose a health risk to unwrapped foods. On the contrary, a significant majority always choose to continue with their chores, not notifying their supervisors of injuries. A large portion (74%) of the participants ensured they washed hands before handling food, while on the other hand, a slightly high proportion of the participants failed to refrigerate leftover food. This was partly because the majority of the students in Kenyan universities prepared their meals in environments that were initially not designated for food preparation such as hostel rooms.

Further, 70% of the participants stored raw food away from cooked foods. According to Oakenfull and Wilson (2020), consumption of infected food originating from animals or cross contaminated foods could potentially lead to the transmission of food borne SARS‐CoV‐2, hence calling for more caution among food handlers. Negligence among university students on matters regarding removal of jewelry before cooking, ensuring the cleanliness of utensils and kitchen counter before and after cooking, could pose a risk to the young food handlers. Despite the coronavirus not surviving cooking, it has a reputation of surviving unwashed surfaces for substantial periods longer than similar viruses (BundesinstitutfürRisikobewertung 2020). Therefore, adhering to stringent measures such as proper scrubbing of fruits and vegetables especially if eaten uncooked, disinfection of utensils, pots, and counter tops at every use should be fiercely embraced (Kramer et al., 2020).

The study affirmed that there was significant correlation between food safety and sanitation knowledge and practice. Particularly, Kenyan female university students had more knowledge and practiced more food safety and sanitation in comparison with their male counterparts. The study findings were interestingly similar to Osaili, Alaboudi, et al. (2011), Osaili, Obeidat, et al. (2011) who also reported that female college students in Jordan were more knowledgeable and less skeptical on practicing, the prevention of cross‐contamination and disinfection procedures. Unklesbay et al. (1998) further reiterated that female students had a significantly (<0.05) higher level of knowledge and practice toward food safety and sanitation compared with male students. The study further observed a significant correlation between attitude and food safety and sanitation. The study implied that the younger categories of university students (20–24 years) had a more positive attitude toward food safety and sanitation compared with the older (30–34 years) categories The study also shows that there is a significant correlation between the types of courses taken in relation to food safety and sanitation knowledge, attitude, and practice. Majority of the students who took science‐based courses such as Consumer Science, Environmental studies, Natural Resource Management, and civil engineering, demonstrated higher levels of knowledge, attitude, and practice toward food safety and hygiene. Similar results were reported by Chuck et al. (2016), who found that some of the highest‐scoring students on food safety knowledge and practice among undergraduates in the University of Maine, were from the faculties of Natural Sciences, Forestry and Agriculture including Food Science and Human Nutrition, Sustainable Agriculture, Biology, and Marine Science. In addition, Ncube et.al (2020) who found that dietetic students in a university in Malaysia had much more knowledge, better attitude, and higher levels of practice in comparison with the institution's food handlers, attributed to the science‐based course they were undertaking.

## 5 CONCLUSION

The study revealed that Kenyan university students have adequate knowledge levels toward food safety and sanitation although have a less satisfactory attitude level. Despite knowledge being the biggest contributor to the practice of food safety and sanitation, many students fail to practice proper food safety and sanitation, which can be attributed to the poor levels of attitude demonstrated by the students toward food safety and sanitation. Kenyan universities should consider introducing food safety courses that emphasize FSMS and HACCP practices and procedures especially to non‐science‐based courses, in various universities to not only reduce the risk of food‐borne diseases but also prevent the spread of COVID‐19. Kenyan universities should provide their students with proper cooking and food handling facilities, to enable adequate preparation and handling of food in a safe and conducive environment, which is sustainable.

## CONFLICT OF INTEREST

All authors declare no conflict of interest in this article.

## AUTHOR CONTRIBUTIONS

**Kevin Serrem:** Conceptualization (equal); Data curation (equal); Formal analysis (equal); Resources (equal); Software (equal); Validation (equal); Visualization (equal); Writing‐original draft (equal). **Csaba Bálint Illés:** Conceptualization (equal); Funding acquisition (equal); Methodology (equal); Project administration (equal); Supervision (equal); Validation (equal); Writing‐review & editing (equal). **Charlotte Atsango Serrem:** Conceptualization (equal); Data curation (equal); Formal analysis (equal); Investigation (equal); Validation (equal). **Bridget Atubukha:** Investigation (equal); Resources (equal); Visualization (equal). **Anna Dunay:** Conceptualization (equal); Methodology (equal); Project administration (equal); Supervision (equal); Visualization (equal); Writing‐review & editing (equal).

## Data Availability

The data that support the findings of this study are available from the corresponding author upon reasonable request.
